# Herpesvirus latent infection promotes stroke via activating the OTUD1/NF-κB signaling pathway

**DOI:** 10.18632/aging.205011

**Published:** 2023-09-09

**Authors:** Jiacai Lin, Yangrui Zheng, Ning Zhao, Fang Cui, Siting Wu

**Affiliations:** 1 Department of Neurology, Hainan Hospital of Chinese PLA General Hospital, Sanya 572013, China; 2 Department of Neurosurgery, Hainan Hospital of Chinese PLA General Hospital, Sanya 572013, China

**Keywords:** cerebral infarction, herpes virus, latent infection, OTUD1/NF-κB signaling pathway, evidence-based medicine meta-analysis, bioinformatics

## Abstract

Objective: Our study aimed to reveal the molecular mechanisms underlying the regulation of cerebral infarction by herpes virus latency infection via the OTUD1/NF-κB signaling pathway using evidence-based medicine Meta-analysis and bioinformatics analysis.

Methods: We conducted a Meta-analysis by searching Pubmed, Embase, and Web of Science databases to evaluate the correlation between herpes virus infection and increased risk of cerebral infarction. We obtained wild-type or mutant herpes virus latent infection-related brain tissue datasets from the GEO database and performed differential analysis to identify differentially expressed genes (DEGs) in the brain tissue after herpes virus latent infection. We further conducted WGCNA co-expression analysis on the cerebral infarction-related datasets from the GEO database to obtain key module genes and intersect them with the DEGs. We used ROC curve analysis to identify the key gene OTUD1 for predicting the occurrence of cerebral infarction and combined correlation and pathway enrichment analyses to identify the downstream pathways regulated by OTUD1.

Results: Our meta-analysis revealed that herpes virus infection is associated with an increased risk of cerebral infarction. By integrating the differential analysis and WGCNA co-expression analysis of GEO chip data, we identified three key genes mediating cerebral infarction after herpes virus latent infection. ROC curve analysis identified the key gene OTUD1, and the correlation and pathway enrichment analyses showed that OTUD1 regulates the NF-κB signaling pathway to mediate cerebral infarction.

Conclusion: Herpes virus latent infection promotes cerebral infarction by activating the OTUD1/NF-κB signaling pathway.

## INTRODUCTION

Cerebral infarction, a type of cerebral vascular disease, affects approximately 13.7 million people annually and is the second leading cause of death [1]. The aging population and the burden of accumulated risk factors have contributed to the increased risk of cerebral infarction, with risk factors including atrial fibrillation, hypertension, hyperlipidemia, hyperhomocysteinemia, diabetes, smoking, physical inactivity, unhealthy diet, abdominal obesity, alcohol consumption [2]. Cerebral infarction is characterized by ischemic necrosis or softening of focal brain tissue caused by obstructed blood supply, ischemia, and hypoxia. Common clinical types of cerebral infarction include cerebral thrombosis, lacunar infarction, and cerebral embolism [3]. Increasing evidence suggests that the secondary progression of cerebral injury is caused by ischemic inflammation, and the severity of the outcome of cerebral infarction depends on the degree of this inflammatory response [4]. Therefore, reperfusion is the main treatment for cerebral infarction, and targeting the inflammatory response after cerebral infarction is currently a challenging and hot topic in clinical research [5].

Herpes simplex virus infection is caused by the DNA virus herpes simplex virus (HSV), which is divided into herpes simplex virus type 1 (HSV-1) and type 2 (HSV-2). HSV-1 primarily causes skin, mucous membranes, and organ (brain) infections outside the genital area, while HSV-2 primarily causes infections of the genital area [6]. Seventy-five percent of viral encephalitis is caused by HSV-1, with neurons in the ganglia being the main site of HSV latency. When latent HSV is activated, it spreads to the temporal and frontal lobes via the axons of the trigeminal nerve. HSV-1 is characterized by its invasion of the temporal lobe, the bottom of the frontal lobe, the insular cortex, and the cingulate gyrus [7]. Some studies have indicated that herpes simplex virus infection is a pathogenic factor of cerebral infarction caused by atherosclerosis. The positive rate of HSV-1 and HSV-2 antibodies was significantly higher in patients with atherosclerosis-related progressive stroke than in non-cerebrovascular disease patients. It provides evidence for the role of herpes simplex virus infection as one of the causes of cerebral infarction, with a close relationship between atherosclerotic cerebral infarction and herpes simplex virus infection [8]. In contrast, cerebral infarction caused by HSV-2 infection is associated with vasculitis causing multiple ischemias, and the prognosis is poor but may be treatable [9].

OTU domain-containing protein 1 (OTUD1) is involved in immune regulation related to infectious diseases [10] and is a biomarker for thyroid cancer. Inhibiting the TGF-pathway inhibitor SMAD7 from cleaving K33-linked polyubiquitin chains and stabilizing the tumor suppressor p53, upregulating the expression of p21 and Mdm2, and speeding up cell apoptosis are all indications that OTUD1 plays a crucial regulatory role in the progression of cancer, antiviral host defense responses, and inflammatory responses [11]. There is also evidence that the loss of OTUD1 is involved in activating the PI3K/AKT and TNF-α/NF-κB signaling pathways by reducing the stability of PTEN [12]. *In vitro* and *in vivo* experiments have shown that OTUD1 expression increases after viral infection, including HSV-1 infection, and OTUD1 knockout enhances the antiviral innate immune response. The increase in OTUD1 expression is established in response to stimuli such as serum deprivation, which can activate NF-κB. The known functions of OTUD1 are concentrated on the DUB activity of target transcription factors, such as the key regulatory molecules p53, YAP, and IRF3 in signaling pathways [13]. It leads us to speculate that OTUD1 may play a role downstream of various biological processes, and while there is currently no direct evidence linking OTUD1 to stroke, it may have some impact on stroke occurrence through NF-κB.

The nuclear factor-κB (NF-κB) transcription factor family is a central factor in the inflammatory process and a key participant in innate and adaptive immune responses [14]. For a long time, the NF-κB pathway has been considered a prototype pro-inflammatory signaling pathway, mainly based on the activation of NF-κB by pro-inflammatory cytokines such as interleukin-1 (IL-1) and tumor necrosis factor α (TNFα), as well as the role of NF-κB in the expression of other pro-inflammatory genes, including cytokines, chemokines, and adhesion molecules [15]. Inflammation is an important issue in stroke treatment, and the harmful effects of inflammation after reperfusion can worsen patient outcomes [16, 17]. The classical NF-κB pathway is activated by the ischemia-induced release of TNF-α, IL-1, IL-6, and other pro-inflammatory cytokines from peripheral immune cells and microglia [18]. NF-κB is the “central hub” of the inflammatory process in the body, with many molecules that interact with NF-κB and subunits that make up NF-κB itself [19], providing many therapeutic targets for inhibiting NF-κB nuclear localization and reducing the inflammatory process of the disease.

This study used evidence-based medicine meta-analysis to find that the risk of stroke increases after herpesvirus infection. Combined with GEO chip differential analysis and WGCNA co-expression analysis, it identified the key gene OTUD1 and its downstream NF-κB signaling pathway associated with latent herpesvirus infection and stroke. This study provides a research foundation for developing drugs for the prevention or treatment of stroke and provides important evidence for the important role of the OTUD1/NF-κB signaling pathway in the recovery process of stroke.

## METHODS

### Database search strategy

We independently searched three English databases, including Pubmed, Web of Science, and EMBASE, by two researchers from their inception to March 2023. Taking Pubmed as an example, a combination of subject headings and free text terms was used for the search. The subject headings mainly included stroke and herpesviruses (including herpes simplex virus types 1 and 2 (HSV-1 and HSV-2), varicella-zoster virus (VZV) or herpes zoster virus (HZ), Epstein-Barr virus (EBV), cytomegalovirus (CMV), herpesviruses 6, 7, and 8) [20].

### Inclusion and exclusion criteria

Cross-sectional research, ecological studies, case series, case reports, and reviews were all eliminated. Studies must report or allow for the derivation of impact estimates. We were not restricted by time, publishing status, language, geographical location, or medical setting. Studies were included if stroke (first or subsequent) was the outcome, either clinically diagnosed or self-reported.

The following are the inclusion criteria: case-control, cohort, or self-controlled case series (SCCS) studies that evaluate the relationship between herpesvirus infection, and the risk of stroke are eligible for inclusion; humans make up the study’s population; the stroke is the outcome; multivariate-adjusted relative risk, hazard ratio, or odds ratio with 95% confidence interval (CI) are provided. Exclusion criteria: (1) duplicate publications; (2) inadequate data; (3) not giving effect sizes (HR, RR, or OR); (3) case studies, analyses, thorough reviews, or abstracts [20, 21].

### Data extraction

A standardized data collection form was used to independently extract data from included studies by two researchers based on the PICOS (population, exposure, comparator, outcome, and study design) framework. If any disputes arose during the data extraction process, they were resolved through discussion and negotiation with multiple research personnel until a consensus was reached. As this was an etiological study, “exposure” replaced “intervention,” and “study characteristics’ were expanded to “study design.” We also recorded the most fully adjusted effect estimate (HR, RR, or OR) for the association between exposure and stroke.

### Evidence-based medicine software and data analysis

The meta-analysis used Stata 12.0 (StataCorp, College Station, TX, USA). We initially used the random-effects inverse variance model to construct the summary OR and its accompanying 95% CI to assess the relationship between herpesvirus exposure and stroke risk. The heterogeneity across studies was measured using the I2 statistic, with larger I2 values suggesting more heterogeneity. After removing trials with significant heterogeneity, the Galbraith plot was used to determine the source of heterogeneity. Using the Metafunnel and Metabias commands, the funnel plot was utilized to examine publication bias subjectively and statistically. The durability of the meta-analysis findings was tested using sensitivity analysis employing the trim-and-fill approach in the presence of publication bias. Additionally, the Metaninf command was used to assess the impact of individual studies on the outcomes of the meta-analysis.

### Gene differential expression analysis

The Gene Expression Omnibus (GEO) database (https://www.ncbi.nlm.nih.gov/gds) was used to obtain the GSE51365 (*Mus musculus*) latent herpesvirus infection-related dataset and the GSE22255 (*Homo sapiens*) blood gene expression dataset for ischemic stroke (IS). The GSE51365 dataset contains brain tissue samples from three control (MOCK), wild-type (MHV-68), and mutant (ORF73) herpesvirus infection groups, respectively. The GSE22255 dataset contains peripheral blood mononuclear cell (PBMC) samples from 20 IS patients and 20 age- and gender-matched controls. The “limma” package in R language filters differentially expressed genes, with a *p*-value < 0.05 as the threshold. The “ggplot2” package was used to plot the gene differential expression volcano plot and box plot, while the “heatmap” package was used to plot the heatmap of differential gene expression. R version 4.2.1 was used for all analyses [22].

### Weighted gene co-expression network analysis (WGCNA)

The R software package “WGCNA” was used to perform WGCNA analysis on the GSE22255 dataset. First, each gene’s median absolute deviation (MAD) was determined, and the bottom 30% of genes with the smallest MAD were removed. Second, the goodSamplesGenes function filters the DEG expression matrix to remove unqualified genes and establish a scale-free co-expression network. Third, a suitable soft threshold was chosen using the “pickSoftThreshold” function; the adjacency matrix was then transformed; the topological overlap matrix (TOM) was then calculated; and finally, a hierarchical clustering dendrogram was built to separate similar gene expression into different modules, with a minimum of 200 genes per module. To merge possible similar modules, a threshold of 0.2 was defined as the cutting height. Finally, the module eigengenes (MEs) were used to summarize the expression profiles of each module, and the correlation between MEs and traits was calculated to select the most relevant modules for further analysis [23–25].

### ROC curve analysis

The significant module genes from the WGCNA analysis were intersected with the differentially expressed genes in the wild-type and mutant strains of murine gammaherpesvirus-infected brain tissues from dataset GSE51365. The results were visualized using the R ggplot2 and VennDiagram packages. The intersection genes were analyzed using the pROC package in R, and the ROC (receiver operating characteristic) curve was plotted using the ggplot2 package [26].

### GO and KEGG enrichment analysis

Pearson correlation analysis was used to screen for genes related to OTUD1 in dataset GSE22255, and the intersection of differentially expressed genes obtained from dataset GSE22255 and the genes related to OTUD1 were visualized using a co-expression heatmap. Further, GO (Gene Ontology) and KEGG (Kyoto Encyclopedia of Genes and Genomes) enrichment analysis was performed using R packages such as “clusterProfiler,” “org.Hs.eg.db,” “enrichplot,” and “GOplot.” The GO and KEGG enrichment analysis results were visualized using a bubble plot [27].

### Statistical analysis

Using STATA 12.0 software, a meta-analysis in evidence-based medicine was carried out. The statistical tests were two-sided, and *P* < 0.05 was considered statistically significant. All statistical analyses in the bioinformatics part were conducted using R software. A non-paired *t*-test was used to compare the two groups, and Pearson correlation analysis was used to observe the correlation between the primary genes and other genes. *P* < 0.05 was considered statistically significant.

## RESULTS

### The meta-analysis of evidence-based medicine shows that there is a correlation between herpesvirus infection and an increased risk of stroke

The meta-analysis included 13 studies (4 case-control studies, 8 cohort studies, and 1 SCCS study) with publication dates ranging from 2007 to 2023. Of these, 8 studies evaluated the relationship between VZV or HZ and stroke, while the other 5 evaluated the relationship between multiple herpesviruses (such as HSV1/2, VZV, EBV, or CMV) infection and stroke. Baseline characteristics of the included literature are presented in the Supplementary File 1.

**Figure 1:**
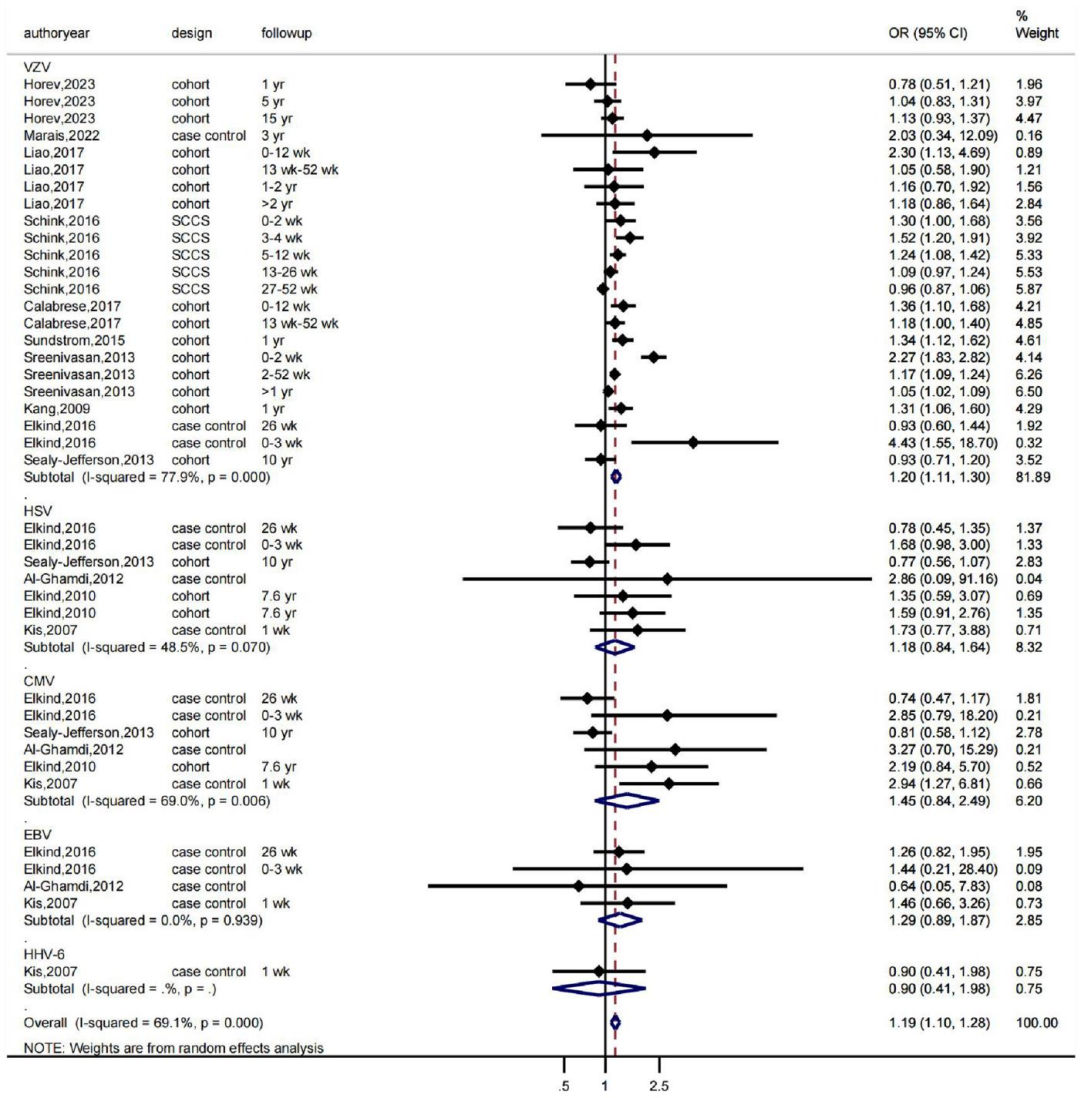
**The effects of herpesviruses, including VZV, HSV, CMV, and EBV, on the risk of stroke.** OR indicates odds ratio, 95% CI indicates 95% confidence interval, and %Weight indicates the weight of each study. The vertical line in the middle represents the null effect line (OR = 1), indicating no statistical association between the studied factor and the outcome. Each horizontal line represents the 95% CI of each study. If the horizontal line of a certain study does not intersect with the null effect line (i.e., the 95% CI does not cross 0), it can be considered that there is a statistical association between the studied factor and the outcome. Abbreviations: yr: represents a year; wk: represents a week.

**Figure 2:**
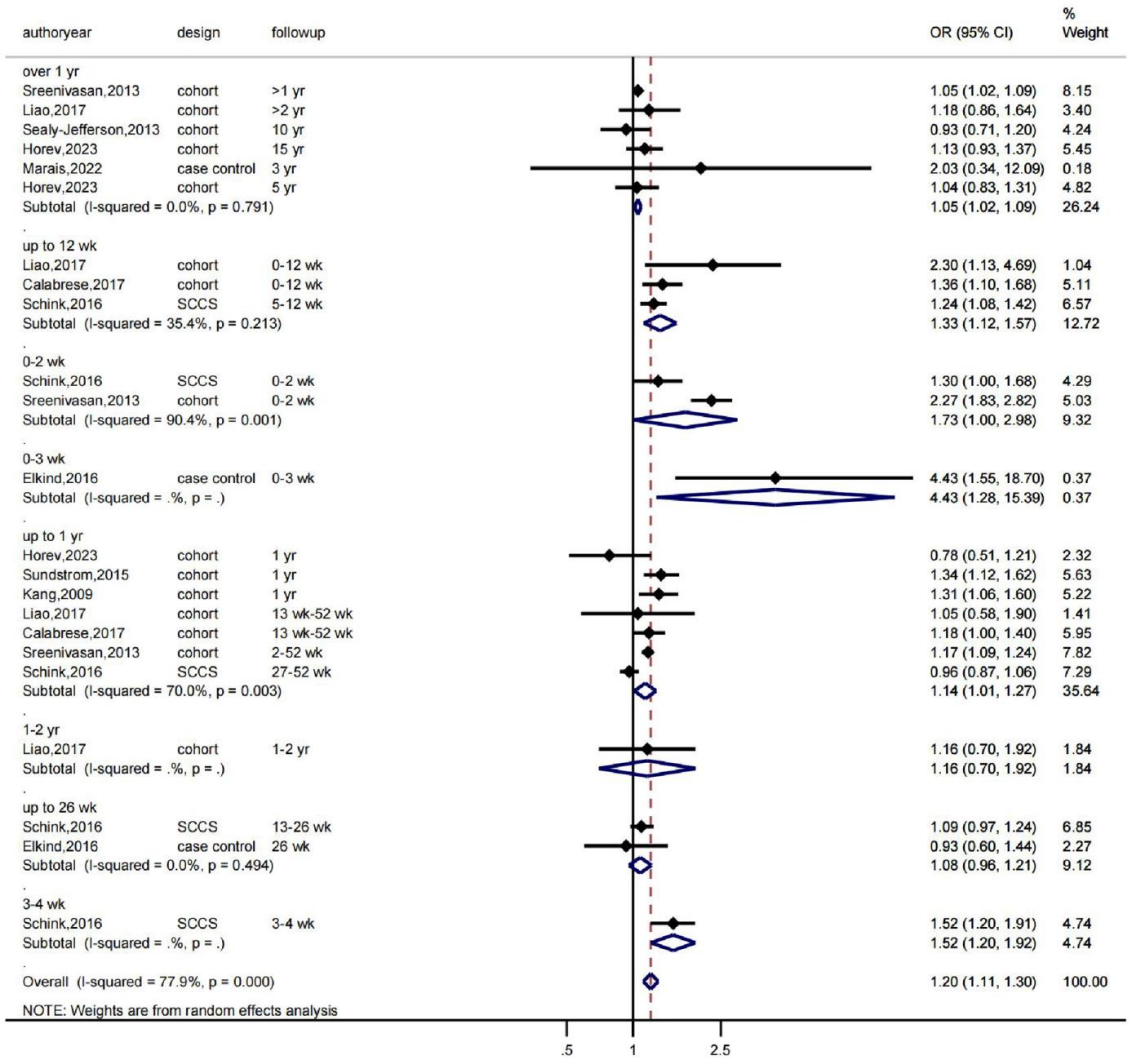
**The effects of VZV infection on the risk of stroke according to follow-up time.** OR indicates odds ratio, 95% CI indicates 95% confidence interval, and %Weight indicates the weight of each study. The vertical line in the middle represents the null effect line (OR = 1), indicating no statistical association between the studied factor and the outcome. Each horizontal line represents the 95% CI of each study. If the horizontal line of a certain study does not intersect with the null effect line (i.e., the 95% CI does not cross 0), it can be considered that there is a statistical association between the studied factor and the outcome. Abbreviations: yr: represents a year; wk: represents a week.

First, we conducted a meta-analysis evaluating the association between various herpesvirus infections and stroke using a random-effects model. The findings revealed that whereas HSV infection was linked to a higher incidence of stroke (OR: 1.18, 95% CI 0.84–1.64, I2 = 48.5%), VZV infection was not (OR: 1.20, 95% CI 1.11–1.30, I2 = 77.9%). While EBV infection was linked to a higher incidence of stroke (OR: 1.29, 95% CI 0.89–1.87, I2 = 0.0%), CMV infection was not (OR: 1.45, 95% CI 0.84–2.49, I2 = 69%) (Figure 1). As shown in Figure 1, there was significant heterogeneity in the VZV group model analysis. Therefore, we further performed a subgroup analysis based on follow-up time, which showed that VZV infection was associated with stroke risk in all follow-up periods, including over 1 year, up to 12 weeks, and up to 26 weeks, and that VZV infection was associated with an increased risk of stroke (Figure 2).

**Figure 3:**
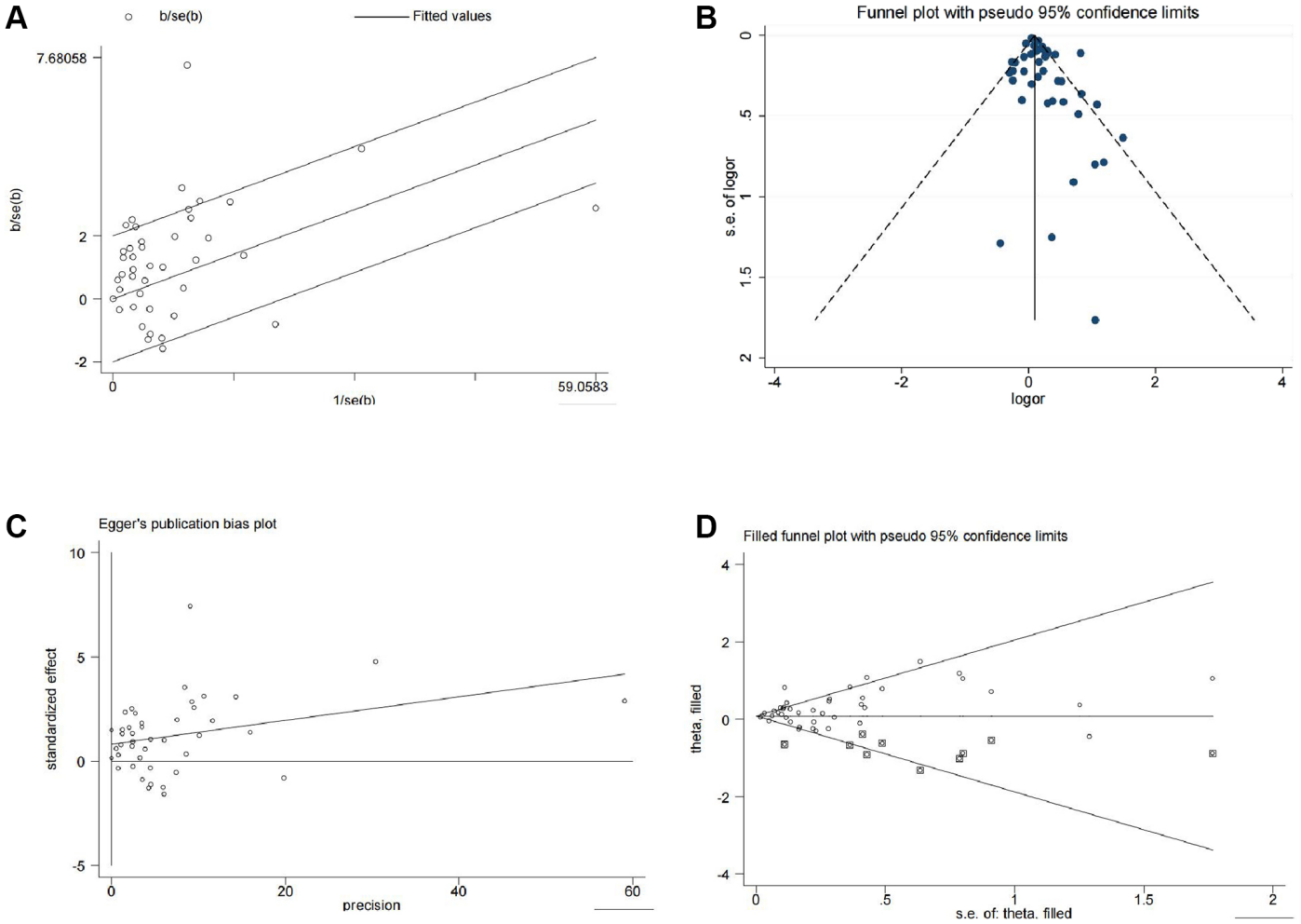
**Heterogeneity, publication bias, and sensitivity analysis of the 13 studies.** (**A**) Galbraith plot for heterogeneity analysis. (**B**) Funnel plot for visual inspection of publication bias. (**C**) Egger’s funnel plot for quantitative evaluation of publication bias. (**D**) Funnel plot for sensitivity analysis using the trim and fill method.

We then conducted further analyses to assess heterogeneity, publication bias, and sensitivity. The Galbraith plot indicated that several studies were outside the confidence interval, suggesting that these studies had larger heterogeneity and may have had a greater impact on the combined effect size (Figure 3A). The funnel plot showed that several studies were not scattered within the funnel (Figure 3B). The Egger’s test indicated a significant asymmetry in the funnel plot with statistical significance (*P* = 0.019) (Figure 3C), indicating the presence of publication bias in the analysis results. Further sensitivity analysis using the trim and fill method showed that the effect sizes (before: fixed: 0.096; random: 0.171; after: fixed: 0.083; random: 0.106) and *P* values (both less than 0.05) changed little, indicating that the meta-analysis results were stable and reliable (Figure 3D). Finally, we examined the influence of individual studies on the results, and the combined effect sizes of each study were within the confidence interval range, indicating that there was little difference among the studies (Supplementary Figure 1).

In conclusion, the evidence-based medicine meta-analysis revealed that herpesvirus infection is linked to a higher risk of stroke, with the strongest associations between VZV, EBV, and HSV infection and stroke risk.

### Identification of differentially expressed genes in wild-type and mutant herpesvirus latent infection-induced brain tissues through GEO chip differential analysis

**Figure 4:**
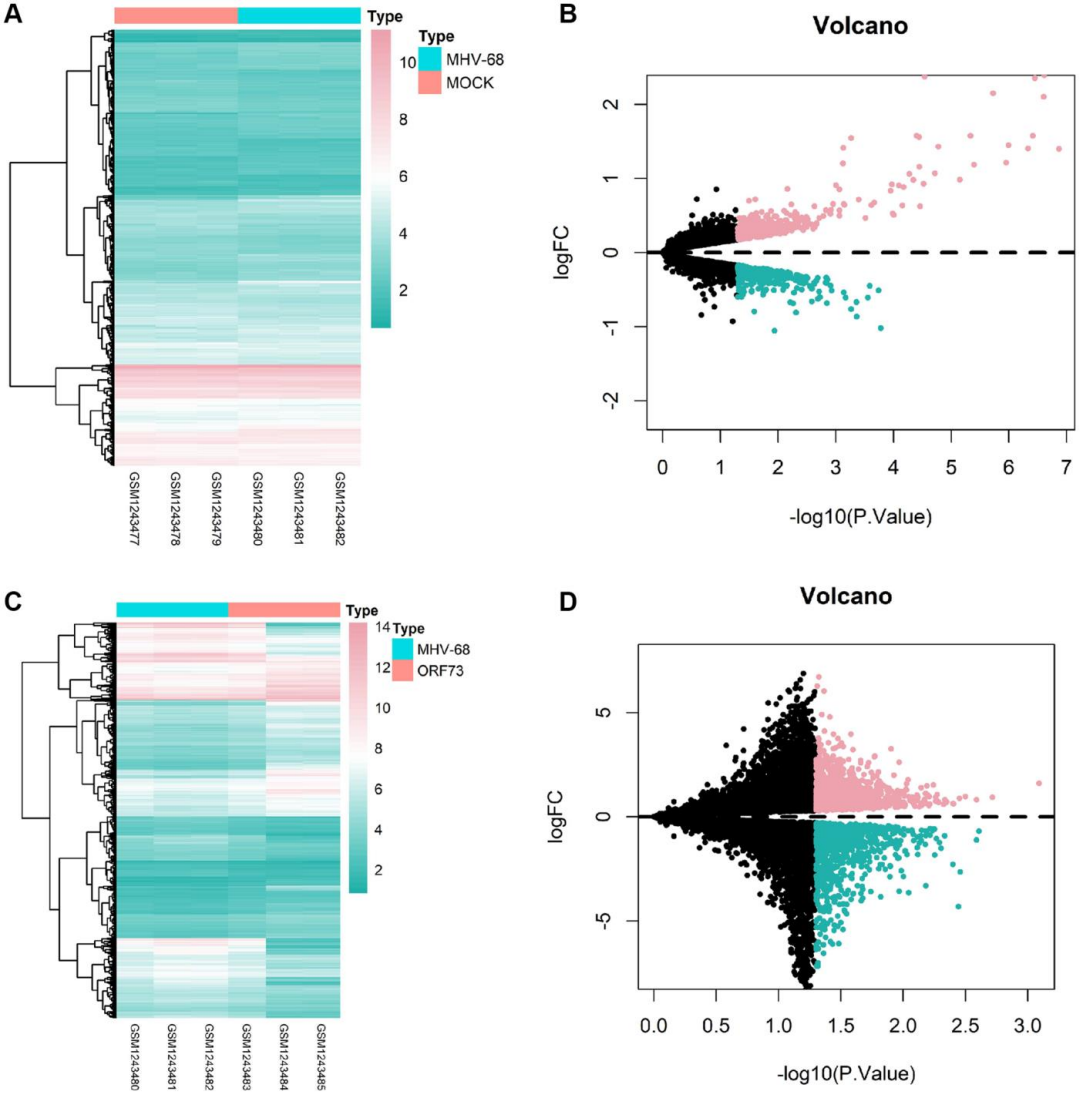
**Heatmaps and volcano plots of differentially expressed genes in brain tissue after latent herpesvirus infection.** Heatmaps and volcano plots of differentially expressed genes in the brain tissue after infection with wild-type herpes simplex virus (HSV)-1 (**A**, **B**) and mutant HSV-1 (**C**, **D**) are shown. In the volcano plots, red dots represent genes with high differential expression, cyan dots represent genes with low differential expression, and black dots represent genes with no differential expression. The MOCK group had a sample size of *n* = 3, the MHV-68 group had *n* = 3, and the ORF73 group had *n* = 3.

To investigate the potential molecular mechanisms underlying herpesvirus latent infection-induced cerebral infarction, we first retrieved the dataset GSE51365 from the GEO database, which contains transcriptome changes induced by latent herpesvirus infection in different organs. We selected brain tissue samples from the control (MOCK) group, wild-type (MHV-68) group, and mutant-type (ORF73) group infected with gamma herpesvirus for differential analysis. We identified 900 differentially expressed genes (449 upregulated and 451 downregulated) in the MHV-68 group (Figure 4A, 4B) and 2104 differentially expressed genes (1142 upregulated and 962 downregulated) in the ORF73 group (Figure 4C, 4D).

### WGCNA co-expression analysis identified 165 stroke-associated hub genes

**Figure 5:**
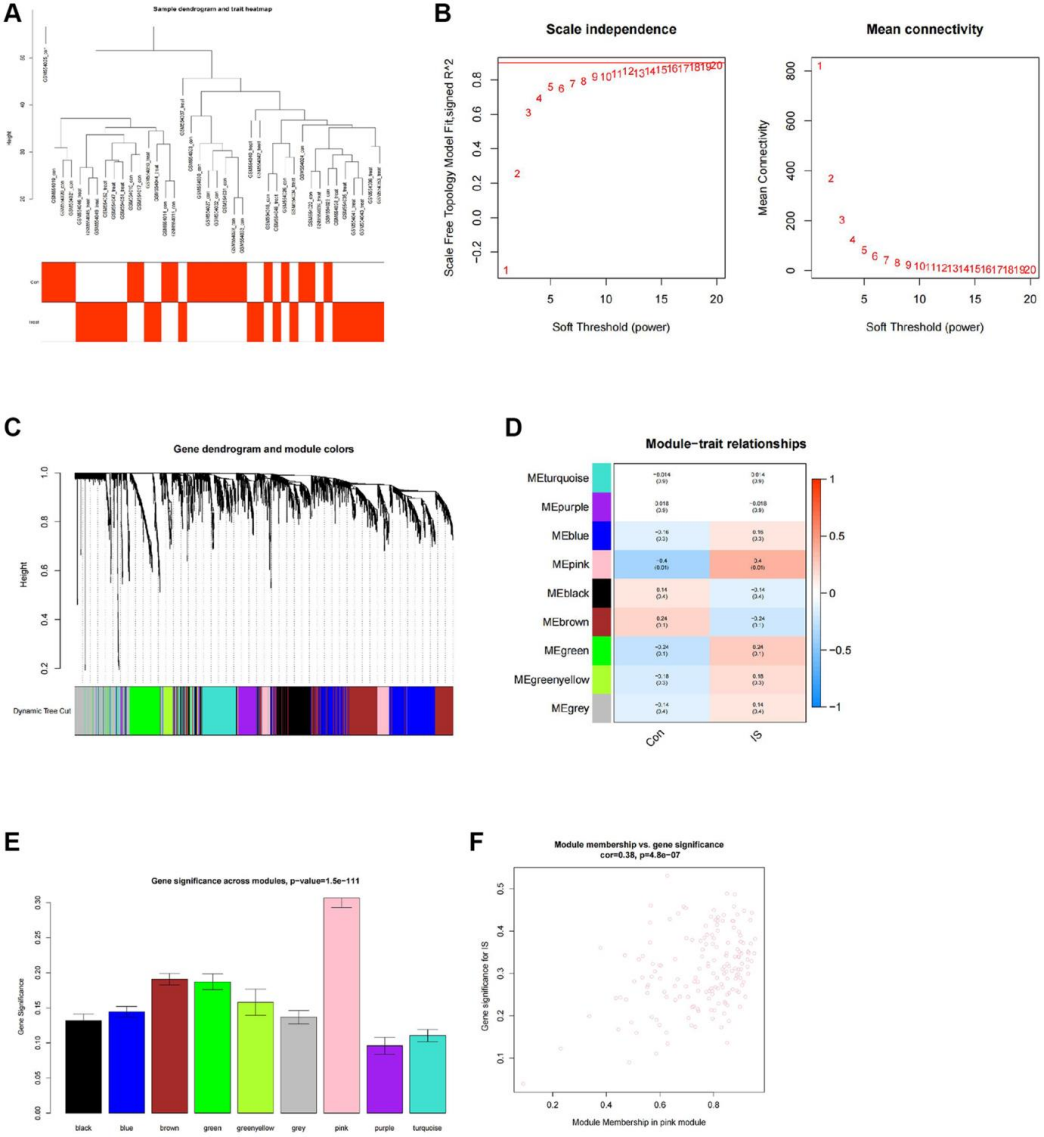
**WGCNA co-expression analysis identifies stroke-related genes.** (**A**) Clustering dendrogram and trait heatmap of control and stroke peripheral blood samples from transcriptomic sequencing dataset GSE22255. (**B**) Scale-free fit index (left) and mean connectivity (right) for various soft-thresholding powers β, with the red line indicating a correlation coefficient of 0.9. (**C**) Clustering dendrogram of co-expressed genes, with each leaf corresponding to a different gene module. (**D**) Heatmap of module-trait correlations in dataset GSE22255, with each cell containing the corresponding correlation and *p*-value. (**E**) Barplot of module gene importance scores. (**F**) Scatterplot of correlation between pink module genes and gene importance. Con (*n* = 20) represents the control group samples, and IS (*n* = 20) represents the stroke group samples.

We further performed a WGCNA co-expression analysis on the GEO stroke-related expression profile dataset. After the hierarchical clustering of the samples (Figure 5A), a soft-thresholding power of β = 12 (scale-free R2 = 0.9) was selected to construct a scale-free network (Figure 5B). Nine gene co-expression modules were identified in the GSE22255 dataset (Figure 5C), among which only the pink module was significantly associated with stroke occurrence with a correlation level of 0.4 (*P* = 0.01) (Figure 5D). The module gene importance was also calculated and revealed that the pink module genes had the highest gene significance score, and their gene importance was significantly positively correlated (Figure 5E, 5F). Therefore, we identified 165 genes in the pink module as stroke-associated hub genes.

### OTUD1 may be a key gene in the mechanism of herpesvirus latent infection-induced stroke

**Figure 6:**
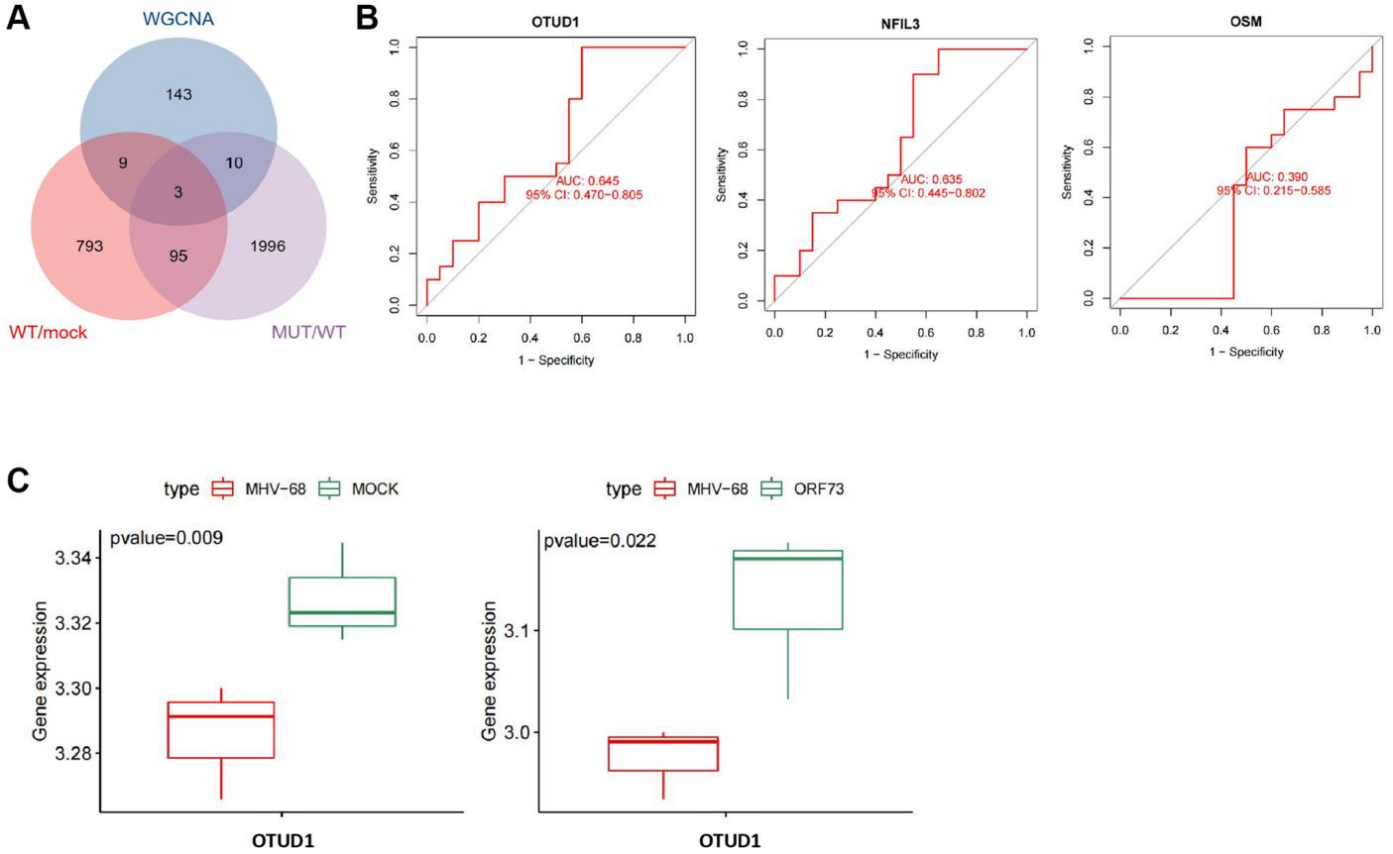
**Key gene selection for herpesvirus latency-mediated stroke occurrence.** (**A**) Venn diagram of the intersection of important module genes identified by WGCNA and differentially expressed genes from wild-type and mutant herpesvirus infection. (**B**) ROC curve analysis of the expression of OTUD1, NFIL3, and OSM genes in relation to stroke disease occurrence. Con (*n* = 20) represents the control group samples, and IS (*n* = 20) represents the stroke group samples. (**C**) Boxplots of differential expression of OTUD1 in brain tissue infected with wild-type and mutant herpesvirus. MOCK (*n* = 3) represents mock-infected brain tissue samples, MHV-68 (*n* = 3) represents wild-type herpesvirus-infected brain tissue samples, and ORF73 (*n* = 3) represents mutant herpesvirus-infected brain tissue samples.

To further identify key genes in the mechanism of herpesvirus latent infection-induced stroke, we took the intersection of the hub genes identified by WGCNA and the differentially expressed genes in wild-type and mutant herpesvirus-infected brain tissues, resulting in three overlapping genes: OTUD1, NFIL3, and OSM (Figure 6A).

ROC curve analysis indicated that OTUD1 and NFIL3 could predict stroke occurrence with a certain degree of accuracy, while OSM could not (Figure 6B). The differential expression of OTUD1, NFIL3, and OSM in the stroke dataset samples is shown in Supplementary Table 1, among which only OTUD1 and OSM were significantly upregulated in stroke. Additionally, differential expression analysis of the GSE51365 dataset revealed that OTUD1 was downregulated in brain tissues infected with wild-type herpesvirus, while it was significantly upregulated in brain tissues infected with mutant herpesvirus (Figure 6C).

Previous studies have reported that after viral infections such as herpes simplex virus 1 (HSV-1) infection, OTUD1 is upregulated, and RNA viruses specifically promote the expression of the deubiquitinating enzyme OTUD1 through an NF-κB-dependent mechanism during the early stages of viral infection [13, 28]. Therefore, we speculate that OTUD1 may be a key gene in the mechanism of herpesvirus latent infection-induced stroke.

### OTUD1 may mediate the occurrence of stroke by activating the NF-κB signaling pathway in herpes simplex virus latent infection

**Figure 7:**
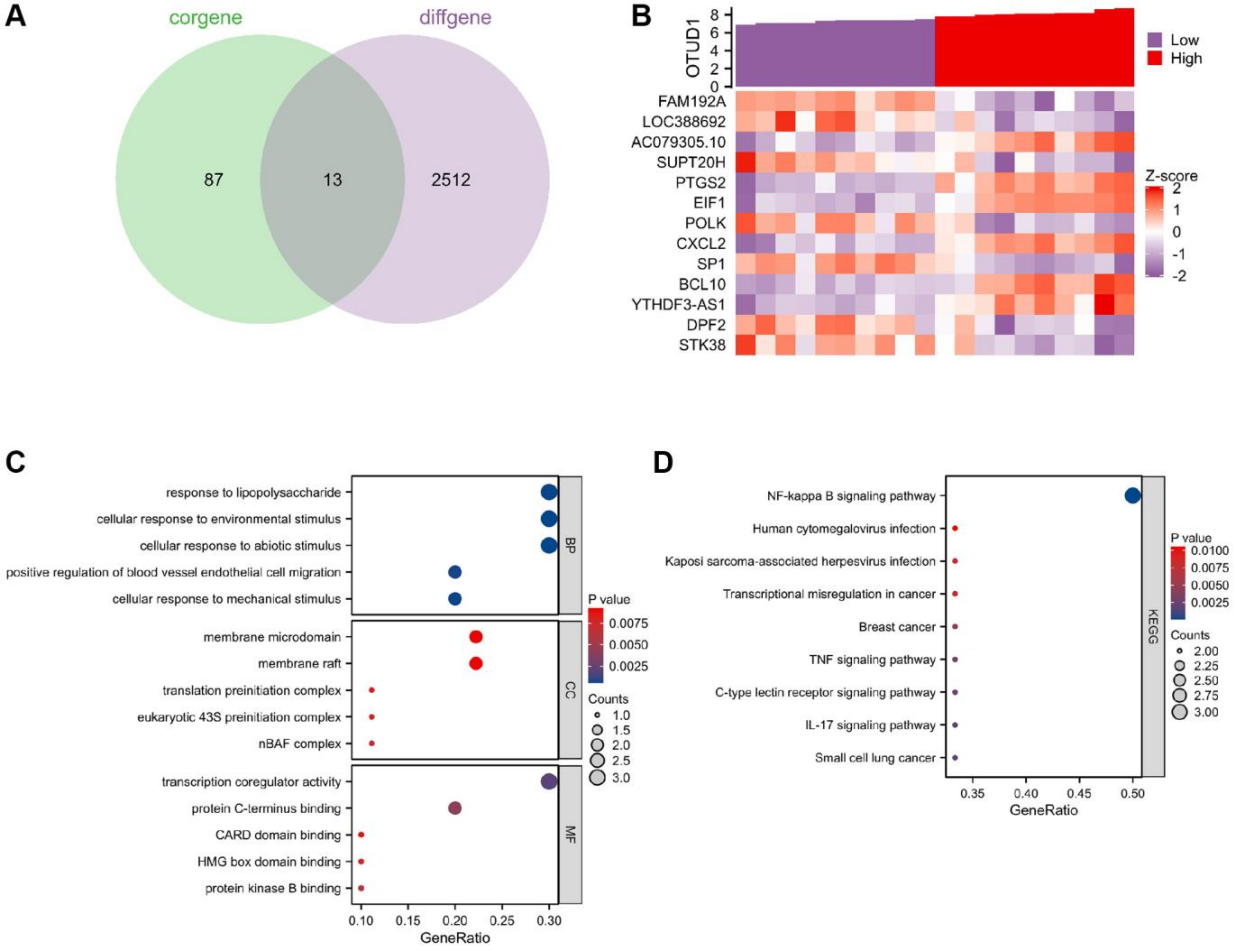
**Key gene selection for herpesvirus latency-mediated stroke occurrence.** (**A**) Venn diagram of the intersection of the top 100 positively and negatively correlated genes with OTUD1 in the GSE22255 dataset and differentially expressed genes in stroke. (**B**) Co-expression heatmap of OTUD1 and 13 intersection genes. The Z-score transformation reduces the visualization effects of large differences in expression values between different variables and preserves differences in expression of individual variables between samples. (**C**) GO functional enrichment analysis of the 14 genes. (**D**) KEGG pathway enrichment analysis of the 14 genes.

To further explore the mechanism by which OTUD1 regulates stroke in herpes simplex virus latent infection, we performed single-gene correlation analysis on the stroke samples in the GSE22255 dataset and obtained 4848 genes significantly positively or negatively correlated with OTUD1. We then intersected the top 50 significantly correlated positive and negative genes with the differentially expressed genes in the stroke samples of the GSE22255 dataset to obtain 13 intersection genes (Figure 7A). The co-expression heatmap of OTUD1 and the 13 intersection genes is shown in Figure 7B, where OTUD1 is significantly positively correlated with PTGS2, CXCL2, YTHDF3-AS1, BCL10, EIF1, and AC079305.10, and significantly negatively correlated with DPF2, POLK, LOC388692, SP1, STK38, SUPT20H, and FAM192A.

Further, GO function and KEGG pathway enrichment analyses of these 14 genes showed that they are mainly involved in the biological processes of forming complexes or complexes with other proteins, participating in lipopolysaccharide reactions, and responding to environmental, mechanical, or non-biological stimuli (Figure 7C). The KEGG analysis showed that these 14 genes are mainly enriched in the NF-kappa B signaling pathway, human cytomegalovirus infection, Kaposi sarcoma-associated herpesvirus infection, IL-17 signaling pathway, and TNF signaling pathway (Figure 7D), indicating that they are closely related to herpes virus infection as well as inflammation and immune response. The most notable enrichment among them is in the NF-B signaling pathway, including the genes PTGS2, CXCL2, and BCL10, which positively correlate with OTUD1. Therefore, we speculate that OTUD1 may mediate the occurrence of stroke in herpes simplex virus latent infection by activating the NF-κB signaling pathway.

## DISCUSSION

HSV-2 infection can cause viral invasion and vascular wall inflammation, which can directly lead to vascular occlusion or secondary thrombus formation, manifested as ischemic stroke, followed by transient ischemic attack (TIA), but without associated intracranial hemorrhage [29]. There is also evidence that varicella-zoster virus (VZV) infection in cerebral arteries leads to vascular pathology that is also a cause of TIA and stroke (Nagel MA., 2014). VZV vascular pathology affects cerebral arteries and can infect extracranial temporal arteries [30]. Current research also points to secondary thrombus development due to viral invasion and arterial wall inflammation as the mechanism of herpes virus infection in the brain, which results in cerebral infarction in individuals with HSV central nervous system infection [31]. In addition, some medical professionals believe that HSV infection of the central nervous system may contribute to posterior circulation stroke in young individuals [32]. Herpes virus-induced cerebral small vessel vasculitis causes endothelial damage, accompanied by secondary bleeding, and immune-mediated inflammatory reactions make brain tissue more prone to bleeding, leading to increased intracranial pressure and increased risk of cerebral infarction [33].

OTU domain-containing protein 1 (OTUD1) is an OTUD subfamily protein containing 481 amino acids and a ubiquitin-interacting motif (UIM) [34]. OTUD1 acts as a DUB to regulate p53 stability and directly inhibits p53 ubiquitination through its catalytic activity. OTUD1 overexpression reduces cell proliferation and increases apoptosis [35]. Although there is no literature data to directly link OTUD1 to cerebral infarction, we have identified OTUD1 as a key gene linking herpes virus latent infection and cerebral infarction. Studies have shown that OTUD1 interacts with NF-κB and interferes with the inflammatory response involved in NF-κB, thereby affecting the homeostatic environment in brain cells. OTUD1 inhibits the activation of nuclear factor kappa B (NF-κB) by removing K63-linked polyubiquitin chains from RIPK1, thereby inhibiting the recruitment of NEMO and the production of pro-inflammatory cytokines, indicating that OTUD1 has an inhibitory effect on NF-κB activation (Wu B., 2022). These findings suggest that OTUD1 may interfere with the recovery process after cerebral infarction by affecting NF-κB, and OTUD1 is also a key target for treating cerebral infarction after herpes virus infection.

The antiviral host defense and inflammatory responses are significantly regulated by OTUD1 [28]. Redox-sensitive transcription factors NF-B and NRF2/KEAP1 collaborate to cause oxidative stress, inflammation, and cell death [36]. These unfavorable outcomes are linked to various illnesses, including neurological and inflammatory disorders (Sivandzade F., 2019). A further crosstalk regulator of the NF-B and NRF2/KEAP1 pathways is OTUD1, a key regulatory component in both signaling pathways. Inhibiting NF-B might lessen tissue damage after an ischemic stroke, enhance neurological prognosis, and perhaps increase the therapeutic window for reperfusion treatment [19]. Furthermore, OTUD1 is an important target for drug development for treating inflammatory bowel disease, hepatitis, sepsis, and kidney cancer due to its association between OTUD1 gene deletion and these disorders [10]. Therefore, identifying an effective inhibitor of the OTUD1/NF-κB signaling pathway to prevent downstream inflammatory and apoptotic signaling is of great significance.

**Figure 8:**
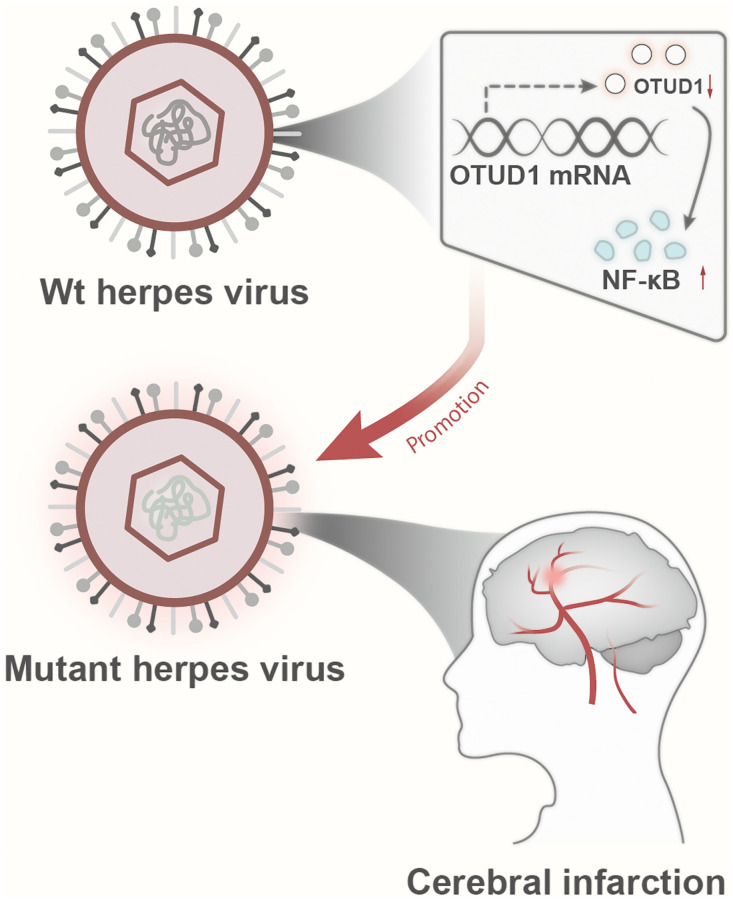
Schematic diagram of the molecular mechanism by which herpesvirus latency activates the OTUD1/NF-κB signaling pathway to promote stroke occurrence.

In conclusion, based on evidence-based medicine meta-analysis and bioinformatics analysis, we found that herpes simplex virus infection may be associated with an increased risk of stroke and herpes simplex virus latent infection promotes stroke occurrence by activating the OTUD1/NF-κB signaling pathway (Figure 8). Through evidence-based medicine meta-analysis and bioinformatics analysis, this research primarily exposes the association between herpes simplex virus infection and stroke and the potential mechanism of herpes simplex virus infection-mediated stroke occurrence. However, *in vitro* and *in vivo* cell and animal experiments have not been conducted to verify the findings; the meta-analysis results are stable. Therefore, we will collect clinical samples from stroke patients in the future to verify the relationship between herpes simplex virus infection and stroke risk and use *in vitro* and *in vivo* experiments to verify the mechanism revealed in this study, if conditions permit, to further explore it.

This study, through meta-analysis, for the first time reveals the relationship between herpesviruses, the OTUD1/NF-κB signaling pathway, and ischemic stroke. This study provides a theoretical basis for the clinical diagnosis and treatment of herpesvirus-induced ischemic stroke while offering potential targets for treating herpesvirus-induced stroke. These findings further enhance our understanding of the occurrence of ischemic stroke. The literature included herpesviruses, including HSV-1/2, VZV, HZ, EBV, and CMV. Based on this, we speculate that the significant findings of this study regarding OTUD1 and the NF-κB signaling pathway may be involved in the risk processes of all viral infections leading to stroke.

## Supplementary Materials

Supplementary File 1

Supplementary Figure 1

Supplementary Table 1
