# Supplementary material for: Herpesvirus latent infection promotes stroke via activating the OTUD1/NF-κB signaling pathway
Source: Aging (Albany NY). 2023 Sep 9;15(17):8976–92. doi: 10.18632/aging.205011 (PMC10522389; doi:10.18632/aging.205011)
Supplement: Supplementary Figure 1 [file aging-15-205011-s002.pdf]

SUPPLEMENTARY FIGURE

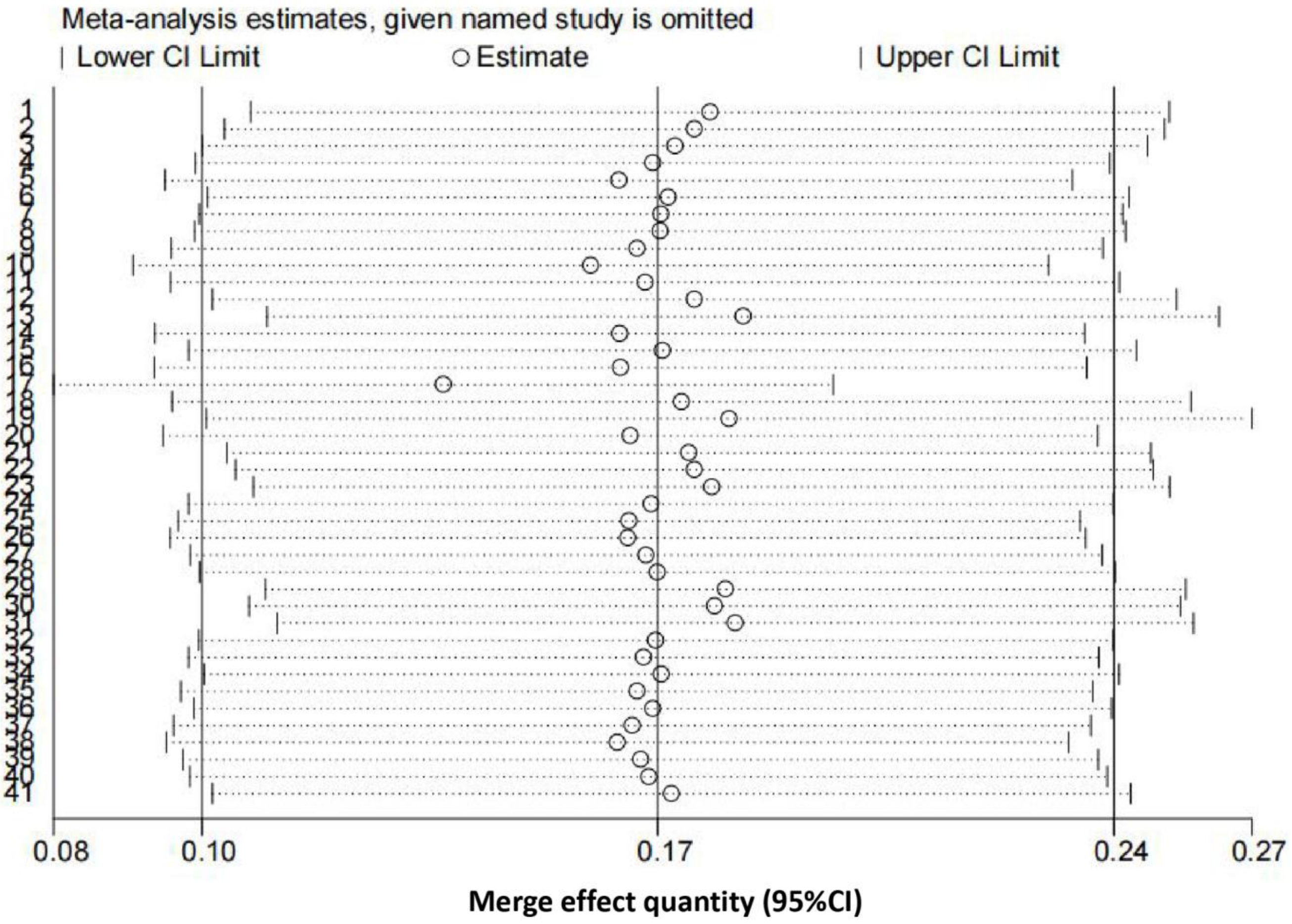

Supplementary Figure 1. Influence of individual studies on the meta-analysis results. Estimates within the confidence interval (0.10–0.27) indicate that the study has little difference from other studies. In all figures, OR indicates odds ratio, 95% CI indicates 95% confidence interval, %Weight indicates the weight of each study, and the vertical line in the middle of the figure represents the null line, OR = 1, indicating no statistical association between the studied factor and outcome. Each horizontal line represents the 95% CI of a given study, and if the line does not cross the null line (i.e., 95% CI does not cross 0), it indicates a statistically significant association between the studied factor and outcome. Abbreviations: yr: indicates a year; wk: indicates a week.
