# Supplementary material for: Herpesvirus latent infection promotes stroke via activating the OTUD1/NF-κB signaling pathway
Source: Aging (Albany NY). 2023 Sep 9;15(17):8976–92. doi: 10.18632/aging.205011 (PMC10522389; doi:10.18632/aging.205011)
Supplement: Supplementary Table 1 [file aging-15-205011-s003.pdf]

SUPPLEMENTARY TABLE

Supplementary Table 1. Differential expression of intersection genes in cerebral infarction dataset GSE22255.

ID	logFC	*P*. Value
OSM	0.8792385	0.01113832
OTUD1	0.3935552	0.043142295
NFIL3	0.501291	0.113354101
